# Transcriptomic Identification of Wheat AP2/ERF Transcription Factors and Functional Characterization of *TaERF-6-3A* in Response to Drought and Salinity Stresses

**DOI:** 10.3390/ijms23063272

**Published:** 2022-03-18

**Authors:** Yang Yu, Ming Yu, Shuangxing Zhang, Tianqi Song, Mingfei Zhang, Hongwei Zhou, Yukun Wang, Jishan Xiang, Xiaoke Zhang

**Affiliations:** 1 College of Agronomy, Northwest A&F University, Yangling, Xianyang 712100, China; yuyang0319@nwafu.edu.cn (Y.Y.); nwafuyuming@163.com (M.Y.); zsx18211672769@163.com (S.Z.); songtiq@163.com (T.S.); zhouhw12356@163.com (H.Z.); wangyukun45@126.com (Y.W.); 2 Academy of Agricultural Sciences, Key Laboratory of Agro-Ecological Protection & Exploitation and Utilization of Animal and Plant Resources in Eastern Inner Mongolia, Chifeng University, Chifeng 024000, China; zhangmingfei0207@163.com

**Keywords:** wheat, AP2/ERF, *TaERF-6-3A*, drought, salt

## Abstract

AP2/ERF (APETALA2/ethylene responsive factor) is a family of plant-specific transcription factors whose members are widely involved in many biological processes, such as growth, development, and biotic and abiotic stress responses. Here, 20 *AP2/ERF* genes were identified based on wheat RNA-seq data before and after drought stress, and classified as AP2, ERF, DREB, and RAV. The analysis of gene structure revealed that about 85% of *AP2/ERF* family members had lost introns, which are presumed to have been lost during the formation and evolution of the wheat genome. The expression of 20 *AP2/ERF* family genes could be verified by qRT-PCR, which further supported the validity of the RNA-seq data. Subsequently, subcellular localization and transcriptional activity experiments showed that the ERF proteins were mainly located in the nucleus and were self-activating, which further supports their functions as transcription factors. Furthermore, we isolated a novel *ERF* gene induced by drought, salt, and cold stresses and named it *TaERF-6-3A*. *TaERF-6-3A* overexpression increased sensitivity to drought and salt stresses in *Arabidopsis*, which was supported by physiological and biochemical indices. Moreover, the expression of stress- and antioxidant-related genes was downregulated in *TaERF-6-3A*-overexpressing plants. Overall, these results contribute to the further understanding of the *TaERF-6-3A* gene function in wheat.

## 1. Introduction

Crop yields are often adversely affected by harsh environmental conditions, including drought, salinity, cold, and high temperatures [1]. Therefore, it is important to prioritize the improvement of crop stress tolerance based on the present and future effects of climate change. There are similarities and differences in the effects of drought and salt stresses. For drought stress, the primary effect is osmotic, whereas salt stress occurs via both osmotic and ionic effects on plant cells [2]. In addition, osmotic stress caused by drought and salt stresses can also rapidly increase the abscisic acid (ABA) content in plants, which induces numerous adaptive responses [3,4]. Plants have evolved protective mechanisms at the molecular, cellular, physiological, and biochemical levels to resist abiotic stress conditions. As master regulators, many transcription factors (TFs) associated with stress tolerance, including AP2/ERF, WRKY, NAC, and MYB, have been recognized as playing crucial functions in various stress response processes in plant-wide stress-response systems [4]. Overexpression of these TF genes increases or decreases adaptation to drought and salt stress. For example, overexpression of *ZmMYB3R* and *SlNAC8* enhances drought and salt resistance in *Arabidopsis thaliana*, whereas overexpression of *GbWRKY1* plays opposite roles in drought and salt stress tolerance [5,6,7]. Therefore, transcription factors play various roles in the plant response to abiotic stress.

The APETALA2/ethylene responsive factor (AP2/ERF) TFs are a large family with one–two well-conserved AP2 domains, and the members of the AP2/ERF family can be divided into three categories, according to the number of AP2/ERF domains and sequence similarity, as AP2, ERF, and RAV (related to ABI3/VP1) [8]. The AP2 subfamily TFs contain two duplicated AP2/ERF domains, and can be further subdivided according to their phylogeny into the AP2 and AINTEGUMENTA (ANT) groups [9]. The RAV subfamily TFs have an AP2/ERF domain and an extra B3 DNA-binding domain, whereas the ERF subfamily TFs only have a single AP2/ERF domain. Sakuma et al. analyzed the phylogenetic relationships and conserved domains in the *Arabidopsis* ERF family, which allowed to further subdivision into three groups of ERF, dehydration-responsive element-binding (DREB), and Soloist [10]. Proteins encoded by the ERF subfamily TFs bind to the AGCCGCC (GCC-box) sequence [11,12], whereas the DREB subfamily TFs containing C-repeats interact with the core *cis*-acting element, CCGAC (DRE/CRT) [13]. ERF subfamily TFs have conserved amino acids at positions 14 (alanine, A) and 19 (aspartic acid, D) of the AP2 domain, and the DREB subfamily TFs have other amino acids also conserved at positions 14 (valine, V) and 19 (glutamic acid, E) [14].

The AP2/ERF TF family has well-studied roles in multitudinous developmental processes in plants. For example, in *Arabidopsis thaliana*, *TaAP2-10-5D* is involved in controlling cell proliferation, which in turn affects organ development, such as floral organ patterning and root and leaf growth [15]. In soybean [16], rice [17], wheat [18], and *Arabidopsis* [19], some AP2/ERF superfamily TFs are involved in yield traits. Meanwhile, the roles of the AP2/ERF family TFs are known to regulate various stress responses, especially the ERF and DREB subfamilies. Several TFs of the ERF and DREB subfamilies have been isolated and identified to be viable candidate genes for enhancing abiotic stress tolerance in plants, including *AtDREB1* (drought) [20], *TaERF1* (salt) [21], *PtERF109* (cold) [22], and *ZmDREB2A* (heat) [23]. Although most ERFs and DREBs function as positive regulators of abiotic stress, others have been found to serve as stress-tolerance repressors that downregulate the level of stress-induced gene transcripts [24,25]. Evidently, the AP2/ERF transcription factor family plays important roles in the responses to abiotic stress in plants.

Due to the complexity of the wheat genome and the fact that the AP2/ERF family is one of the largest transcription factor families in plants, *AP2/ERF* members are abundant in wheat, and their functions also appear to be diverse. In other words, it is difficult to quickly and uniquely determine the specific *AP2/ERF* genes associated with abiotic stress. Transcriptome sequencing has the potential to rapidly identify and characterize novel genes related to abiotic stress. Therefore, transcriptome sequencing was used to quickly and accurately identify the *AP2/ERF* family genes that might correspond to stress resistance. In this study, a total of 20 *AP2/ERF* genes were identified in an RNA-seq database of differentially expressed genes (DEGs) in wheat, before and after drought stress. We comprehensively analyzed the phylogenetics, gene structures, expression levels, subcellular localizations, and self-activation activity of the *TaAP2/ERF* genes in wheat. Transgenic *Arabidopsis* overexpressing *TaERF-6-3A* displayed hypersensitivity to drought and salt stress. Furthermore, we also found that TaERF-6-3A interacts with the transcription factor AtCIPK22 in yeast two-hybrid assays. Overall, our results enhance the understanding of the AP2/ERFs in wheat, providing valuable clues for the functional analysis of the wheat *AP2/ERF* family genes.

## 2. Results

### 2.1. Transcriptomic Identification of AP2/ERF Genes in Wheat

**Figure 1:**
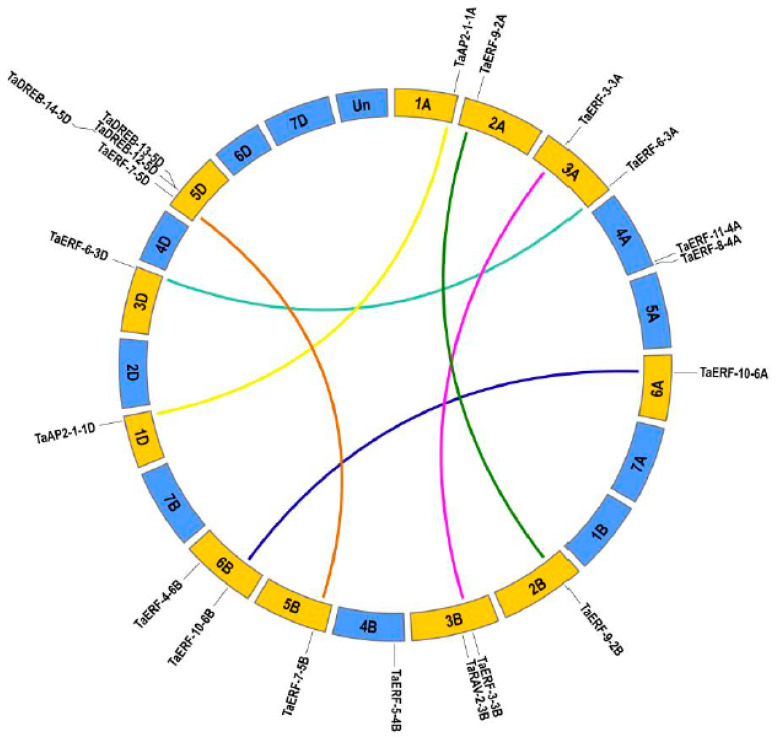
Chromosome distribution of *TaAP2/ERF* genes. Colorful lines indicate homologous copies (distributed among A, B, or D sub-genomes).

**Figure 2:**
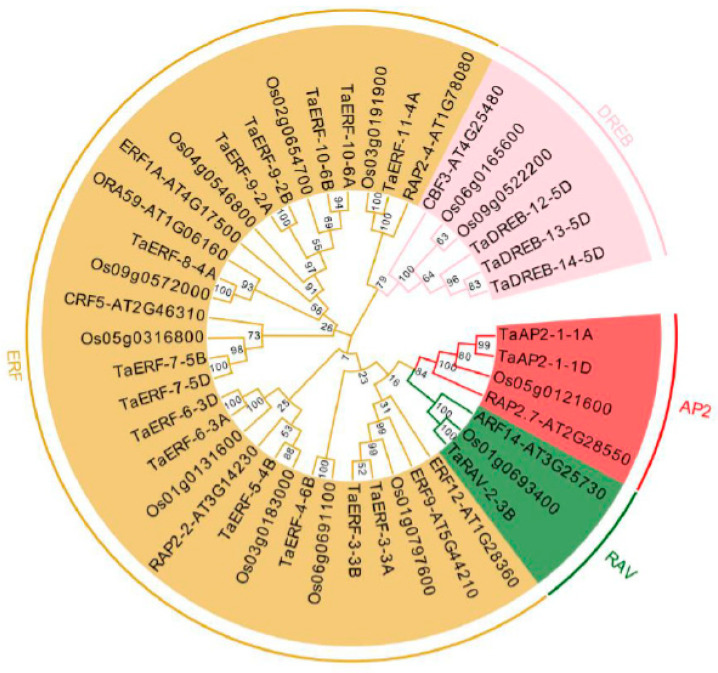
Phylogenetic relationships of wheat, rice, and *Arabidopsis* AP2/ERF proteins. The neighbor-joining tree was divided into four groups (AP2, RAV, ERF, and DREB).

Differentially expressed genes (DEGs) were characterized (FDR < 0.05 and a fold change ≥ 2) and were identified in wheat before and after drought stress. A total of 2381 DEGs were found, of which 1339 were up-regulated and 1042 down-regulated (Figure S1 and Table S1). To identify *AP2/ERF* genes in wheat, a transcriptome-wide analysis was operated by local BLASTP using the wheat E2F/DP HMM profiles. After examining all putative DEGs using the NCBI CD-search and manual regulation, a total of 20 *AP2/ERF* members were identified as AP2/ERF TFs in an RNA-seq database of differentially expressed genes (DEGs) in wheat before and after drought stress (Table S3). The chromosome distribution results indicate that these *TaAP2/ERF* genes are located on 13 of the 21 chromosomes in wheat. *TaAP2/ERF* genes were most abundant on chromosome 5D, with four *TaAP2/ERF* genes, whereas eight *TaAP2/ERF* genes were evenly distributed over chromosomes 3A, 3B, 4A, and 6B. The remaining eight *TaAP2/ERF* genes were singly distributed on eight other chromosomes (Figure 1). These *TaAP2/ERF* genes were renamed according to their chromosomal locations and evolutionary relationships (Table S3 and Figure 2). We analyzed the characteristics of the TaAP2/ERF proteins, including the amino acid length, isoelectric point (PI), molecular weight (MW), and predicted subcellular localization (Table S3). Accordingly, the lengths of the 20 TaAP2/ERFs were found to range from 212 aa (TaDREB-12-5D) to 510 aa (TaAP2-1-1D), PI values ranged from 4.76 (TaERF-6-3A) to 9.24 (TaERF-3-3B), and the MWs varied from 23.34 KDa (TaDREB-12-5D) to 55.49 KDa (TaAP2-1-1A). In addition, the prediction of subcellular localization showed that most (18/20, 90%) TaAP2/ERF proteins localized in the nuclear region, whereas the remainder (2/20, 10%) localized to the plasma membrane.

### 2.2. Phylogenetic Analysis of TaAP2/ERFs

A total of 20 wheat AP2/ERFs, 13 rice AP2/ERFs, and 10 *Arabidopsis* AP2/ERFs were used to generate an NJ phylogenetic tree (Figure 2). All 20 members of the TaAP2/ERF family were classified into AP2, ERF, DREB, and RAV subfamilies, among which the ERF subgroup was the largest (14 members) and the RAV subgroup was the smallest (one member). The TaAP2/ERF proteins were clustered together with proteins that were derived from monocot rice AP2/ERFs and clustered separately from the dicotyledonous *Arabidopsis* AP2/ERFs.

### 2.3. Structural Analysis of TaAP2/ERFs

**Figure 3:**
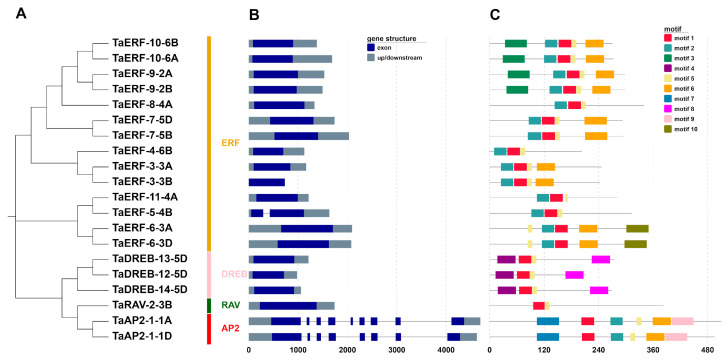
TaAP2/ERF family members analyzed according to (**A**) phylogenetic relationships, with division into four groups (AP2, RAV, DREB, and ERF); (**B**) gene structure, where exons and introns are indicated by dark blue boxes and dark gray lines, respectively; and (**C**) motif composition, with the 10 conserved motifs indicated with boxes of different colors.

Intron–exon structures are helpful for understanding the structural characteristics of the TaAP2/ERF family (Figure 3A,B). The number of introns varied in the different subfamilies. ERF, DREB, and RAV subfamilies in wheat contained no or one intron, whereas the members of the AP2 subfamily had seven to eight introns. Most TaAP2/ERFs (17/20, 85%) were intronless, except in the case of three genes (*TaAP2-1-1A*, *TaAP2-1-1D*, and *TaERF-5-4B*) whose intron numbers ranged from one to eight. Additionally, the distribution of introns also showed interesting differences among diverse subfamilies. Concerning the *TaAP2/ERF* sequences containing introns, the introns were mostly located near the N-termini (*TaERF-5-4B*) or C-termini (*TaAP2-1-1A* and *TaAP2-1-1D*) and rarely in the middle of the sequences. The high variation in the gene structure reveals that there was widespread differentiation during the formation and evolution of the wheat *TaAP2/ERF* genome.

Furthermore, the TaAP2/ERF proteins were analyzed for the presence of conserved domains (Figure S2). The proteins encoded by *TaAP2/ERF* genes have a highly conserved AP2/ERF domain. AP2 subfamily proteins contain two AP2 domains in series, whereas ERF and DREB subfamily proteins contain only a single AP2 domain. TaRAV-2-3B from the RAV subfamily contains an additional B3 domain consisting of 106 amino acids, in addition to an AP2 domain (Figure S2).

The presence of conserved motifs was further analyzed using MEME online software (Figure 3C). A total of 10 motifs were identified and named as motifs 1 to 10 (Figure S5). Among these, motifs 1 and 2 were predominant, being found in the AP2/ERF domains of all TaAP2/ERF TFs (Figure S3). The protein sequences of four members belonging to the AP2/ERF family contained motif 5. Motif 7 and motif 9 were only found in TaAP2 subfamily members, whereas motif 8 was only found in TaDREB members. Additionally, all TaERF and TaDREB protein sequences were identified to have a highly conserved WLG motif (Figure S3). Most (11/14, 78.57%) TaERF proteins were conserved at positions 14 (A, alanine) and 19 (D, aspartic acid), within their AP2/ERF domains (Figure S3).

### 2.4. Cis-Acting Elements of TaAP2/ERF Genes

**Figure 4:**
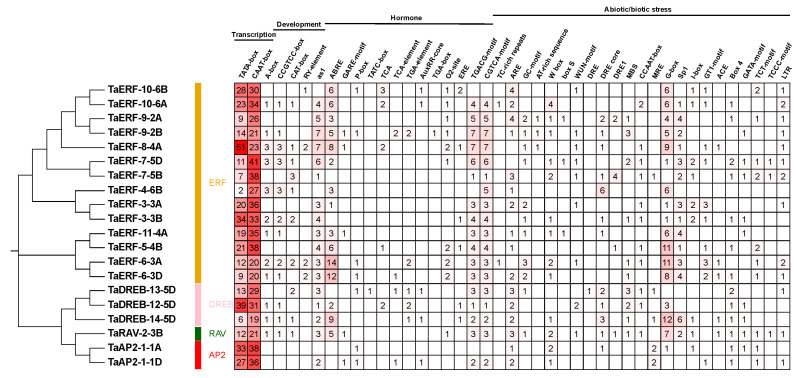
Main predicted *cis*-acting elements in *TaAP2/ERF* promoters. Four types of *cis*-acting elements (transcription-associated; development-associated; hormone-associated; and abiotic stress-associated) were identified in the promoter regions (−2000 bp).

To further reveal the potential functions of *TaAP2/ERF* genes, *cis*-acting elements of these genes were identified using PlantCARE (Figure 4). Many *cis*-acting elements were detected, including transcription-related, development-responsive, hormone-responsive, and abiotic stress-responsive elements. Transcription-related *cis*-elements (TATA-box and CAAT-box) were observed in all the *TaAP2/ERF* genes. Hormone responsive *cis*-elements; ABREs; GARE motifs; P-box; TATC-box; TCA element; TGA element; AuxRR core; TGA box; O2 site; TGACG motifs; and CGTCA motifs accounted for 75; 25; 35; 5; 20; 25; 25; 5; 50; 85; and 90% of *TaAP2/ERF* gene promoters, respectively. Meanwhile, there were many abiotic stress-related *cis*-elements, such as the defense and stress responsiveness element (TC-rich repeats); anaerobic induction element (ARE); WRKY transcription factor binding element (W-box); wound responsiveness element (WUN motif); DRE, drought response element (MBS); and low-temperature responsiveness (LTR). The above information revealed that the *TaAP2/ERF* genes may be regulated through multiple *cis*-acting elements and play vital roles in plant development and stress responses.

### 2.5. Expression Profiles of TaAP2/ERF Genes in Drought Stress

**Figure 5:**
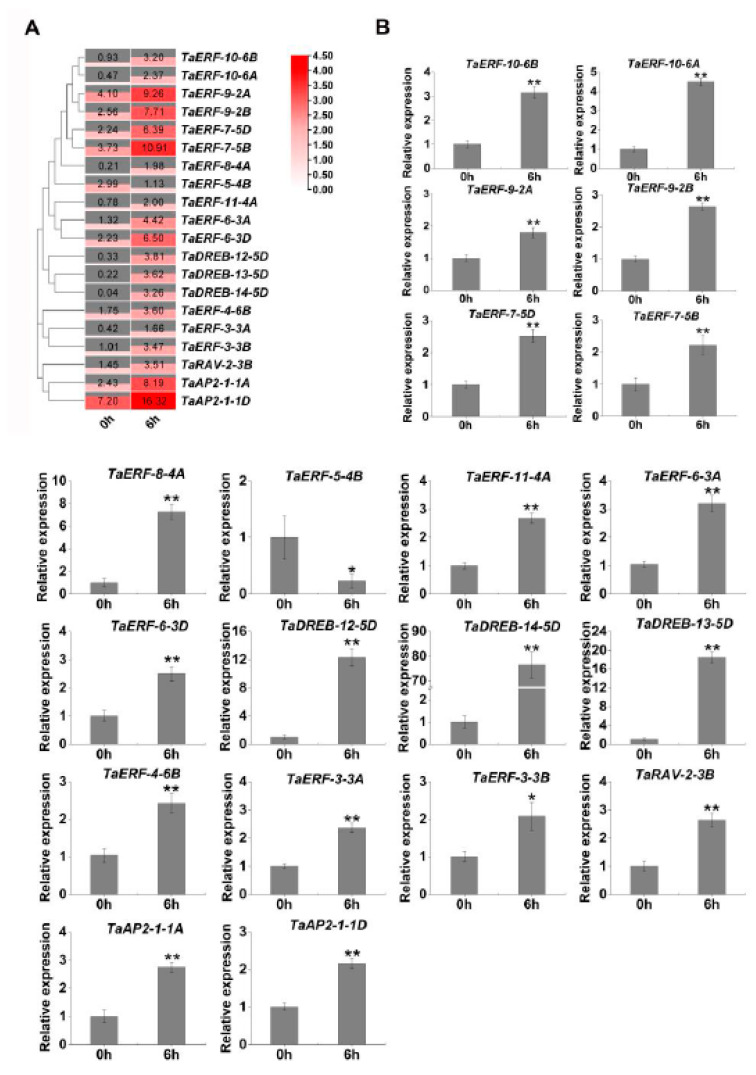
RNA-seq data analysis and qRT-PCR verification. (**A**) Heatmap of 20 *AP2/ERF* gene members in the leaves of JW1 before and after drought stress. The changes in expression levels are visually reflected in the heatmap with the gradient color white (low)/red (high). The heatmap was created using TBtools software. Data are the mean fragments per kilobase million (FPKM) values in RNA-seq data, which reflect the relative transcript abundance; (**B**) Expression profiles of 20 *TaAP2/ERF* genes under drought stress. Columns represent the results of qRT-PCR verification. Standard deviations are indicated by error bars (the mean ± SD, and n = 3), and significantly different values are indicated asterisks (ANOVA, * *p* < 0.05; ** *p* < 0.01).

To further elucidate the biological functions of *TaAP2/ERF* genes, we analyzed the expression profiles of the 20 *TaAP2/ERF* genes identified from our RNA-seq database under drought stress (0 and 6 h) (Figure 5A and Table S4). Almost all DEGs were upregulated under drought stress, except for *TaERF-5-4B*. For most (12/20, 60%) of the TFs, drought stress induced the expression by between 2.5- and 9.5-fold. In particular, the drought-induced gene expression of the TaDREB subfamily TFs was more than 10-fold higher than the initial level. For example, *TaDREB-14-5D* was barely expressed at 0 h and finally sharply increased about 89-fold after 6 h under drought stress. In the AP2/ERF subfamily, the expression of *TaAP2-1-1D* was relatively abundant before and after drought-stress induction, though the magnitude of fold induction was low. The results represent that many TaAP2/ERFs in wheat are involved in drought stress, but that they may be functionally distinct.

To validate the fragments per kilobase million (FPKM) expression profiles of *TaAP2/ERF,* calculated according to the RNA-seq data (Figure 5A), we characterized the expression of 20 *AP2/ERF* genes using qRT-PCR. The verification results were roughly consistent with the RNA-seq results (Figure 5B), which suggest that the RNA-seq data are reliable. The expression patterns of *AP2/ERF* genes were diverse, with *TaERF-5-4B* downregulated and the rest upregulated, suggesting that these genes may have different biological functions. ERF TFs are known to play important roles in response to abiotic stresses. In our study, most of the identified *AP2/ERF* family genes in the wheat RNA-seq database were *ERF* members. Therefore, we randomly selected four upregulated genes (*TaERF-3-3B; TaERF-6-3A; TaERF-7-5D;* and *TaERF-9-2B*) and one downregulated gene (*TaERF-5-4B*) for further analysis.

### 2.6. Subcellular Localization and Transcriptional Activity Analysis

**Figure 6:**
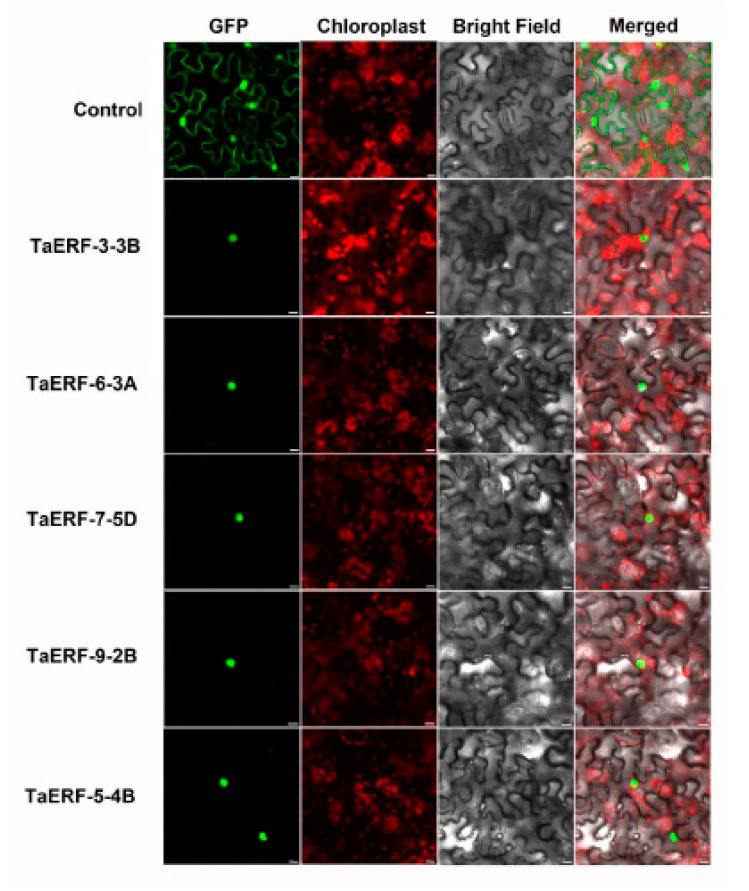
Subcellular localization of five TaAP2/ERF proteins as GFP fusions. Recombinant CaMV35S:TaAP2/ERF-GFP vector and control CaMV35S-GFP vector were transiently expressed in tobacco cells.

As transcription factors, TaAP2/ERF proteins are expected to be localized in the nucleus. Furthermore, our previous prediction results also indicated that the TaAP2/ERF family members localize in the nucleus (Table S3). Thus, to verify these predictions, we first cloned five *TaAP2/ERF* genes (*TaERF-3-3B; TaERF-6-3A; TaERF-7-5D;* *TaERF-9-2B;* and *TaERF-5-4B*) from the wheat variety JW1 and constructed corresponding transient vectors for the expression of each as GFP fusions, which were then separately transformed into *Nicotiana benthamiana* leaf cells. Laser confocal microscopy was used to track the localization of the TaAP2/ERF proteins. As shown in Figure 6, all five proteins were expressed as TaERF-GFP fusion proteins in transformed *Nicotiana benthamiana* leaf cells, and they were mainly localized in the nucleus. The above experimental results are highly consistent with our conjecture and predictions.

**Figure 7:**
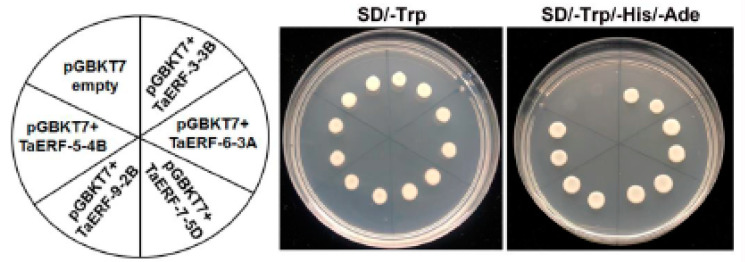
Transcriptional activation of five TaAP2/ERF proteins. The ORF fragments of *TaAP2/ERF* genes were ligated with pGBKT7 to construct a fusion vector. The yeast cells harboring TaAP2/ERF fusion vectors were cultured on the SD/-Trp and SD/-Trp/-His/-Ade.

To detect whether the five full-length TaERF proteins had self-activation activity, PGBKT7-*TaERF* recombinant vectors were transformed into yeast cells for Y2H and screened on the SD/-Trp and SD/-Trp/-Ade/-His yeast media. All yeast colonies with pGBKT7-TaERFs could grow on both SD/-Trp and SD/-Trp/-Ade/-His media (Figure 7), demonstrating that the five TaERF proteins showed self-activation activity.

### 2.7. TaERF-6-3A Expression in Wheat Is Induced by Drought, Salt, and Cold

**Figure 8:**
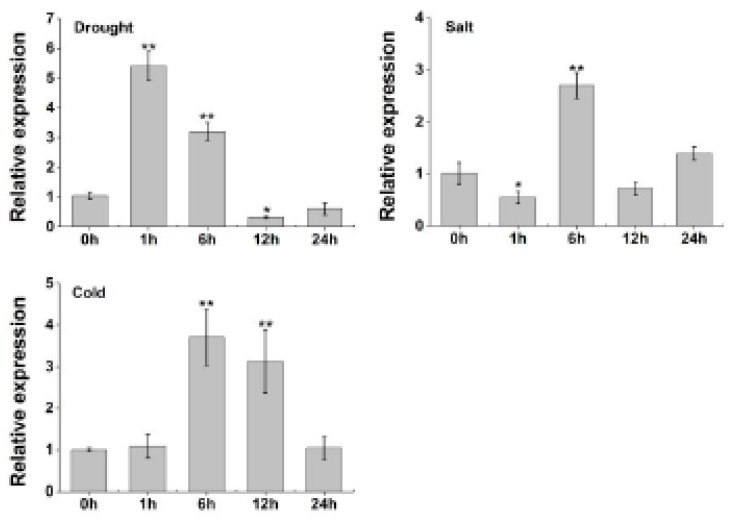
Expression patterns of *TaERF-6-3A* in response to conditions of drought (20% PEG6000), salt (200 mM NaCl), and cold (4 °C) as analyzed by qRT-PCR. Standard deviations are indicated by error bars (mean ± SD and n = 3), and significant differences are indicated using asterisks (ANOVA, * *p* < 0.05; ** *p* < 0.01).

The above *cis*-acting element analysis showed that the promoter of *TaERF-6-3A* contained the most ABREs of the analyzed genes (Figure 4), suggesting that *TaERF-6-3A* may respond to abiotic stress via the ABA pathway. In addition, *cis*-acting elements related to drought induction (MBS) and cold stress (LTR), and the WRKY transcription factor binding element (W-box) were also found in the *TaERF-6-3A* promoter region (Figure 4). The above information suggests that *TaERF-6-3A* may respond to different abiotic stresses. Thus, we selected *TaERF-6-3A* for further investigation. We used qRT-PCR to evaluate the expression levels of *TaERF-6-3A* under a variety of stress conditions, such as drought, salt, and cold (Figure 8). Under drought stress, *TaERF-6-3A* exhibited an initial stress response through rapidly increased expression, which reached a peak at 1 h before decreasing and reaching a minimum 12 h later, with slow recovery to initial levels by 24 h. Under salt stress, *TaERF-6-3A* showed only slight downregulation at 1 h, followed by an upward trend, peaking at 6 h, and subsequently decreasing. Under cold stress, the expression level of *TaERF-6-3A* did not change significantly after 1 h treatment, at which point it proceeded to rapidly increase, peaking at 6 h, then decreased slowly and recovered to the initial level by 24 h. These results show that the expression of *TaERF-6-3A* is responsive to multiple abiotic stresses.

### 2.8. Functional Characterization of TaERF-6-3A in Transgenic Arabidopsis under Drought and Salt Stresses

**Figure 9:**
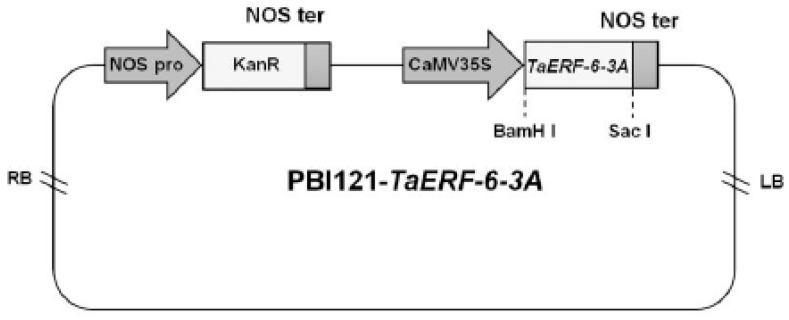
Schematic diagram of plant overexpression vector pBI121. LB, left border; RB, right border. NOS pro, nopaline synthase gene promoter; NOS ter, nopaline synthase gene terminator; CaMV 35S, CaMV 35S promoter; KanR, kanamycin resistance; BamH I and Sac I, enzyme-digested sites.

**Figure 10:**
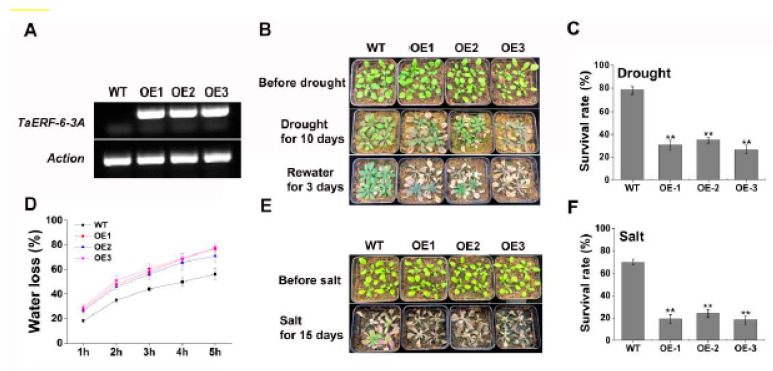
Functional characterization of *TaERF-6-3A* in transgenic *Arabidopsis* under drought and salt stress. (**A**) qRT-PCR verification of *Arabidopsis* overexpression (OE) lines. *TaERF-6-3A* specific primers were used to sense transgene levels. *Arabidopsis Actin* served as an internal control; The (**B**) phenotypes and (**C**) survival rates of *TaERF-6-3A* OE lines after withholding water for 10 days followed by resumption of watering for 3 days; (**D**) Water loss rates of 3-week-old OE lines; The (**E**) phenotypes and (**F**) survival rates of *TaERF-6-3A* OE lines after salt stress for 15 days. Standard deviations are indicated by error bars (mean ± SD and n = 3) and significant differences are indicated using asterisks (ANOVA, ** *p* < 0.01).

In order to reveal the potential function of *TaERF-6-3A*, an overexpression vector for it was constructed (Figure 9), and T_3_ generation transgenic *Arabidopsis* was obtained. The results of RT-PCR show that *TaERF-6-3A* could be detected in *Arabidopsis* overexpression (OE) lines (OE-1, OE-2, and OE-3 line) but not in the wild type (WT) (Figure 10A). This indicates that *TaERF-6-3A* was successfully transformed into *Arabidopsis thaliana*. In drought resistance analysis, 3-week-old *Arabidopsis* plants were not provided water for 10 days, after which most OE lines had severely wilted (Figure 10B). After drought stress for 12 days, and re-watering for 3 days, the survival rates of the transgenic OE lines were only 25–35%, which was significantly lower than for WT lines (78.3%) (Figure 10C). In addition, the rates of water loss were higher for OE than for WT leaves (Figure 10D). In the salt resistance analysis, 3-week-old *Arabidopsis* plants were irrigated with a 300 mM NaCl solution for 15 days. The OE lines became severely wilted and died, whereas the symptoms of the WT lines were relatively mild (Figure 10E). Correspondingly, the survival rates of transgenic OE lines were significantly lower than that of the WT lines (Figure 10F). These results suggest that *TaERF-6-3A* is a negative regulator of drought and salt stress responses.

**Figure 11:**
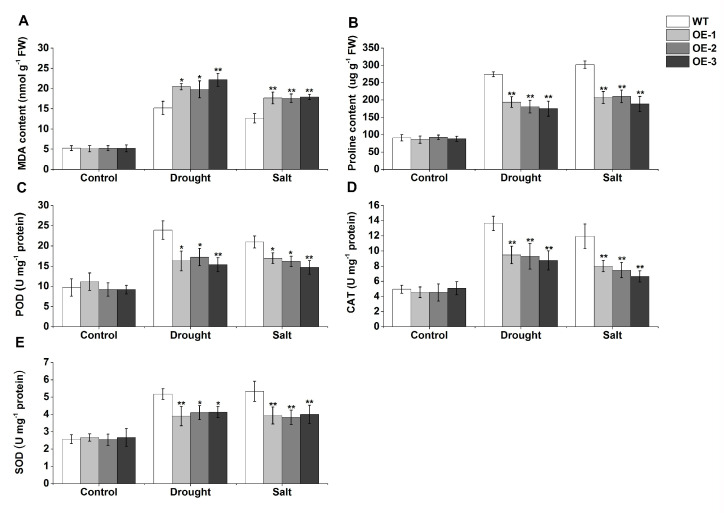
The effect of drought and salt stresses on MDA (**A**); proline (**B**); and the activities of POD (**C**); CAT (**D**); and SOD (**E**) in *TaERF-6-3A* transgenic *Arabidopsis* lines. Standard deviations are indicated by error bars (mean ± SD and n = 3) and significant differences are indicated using asterisks (ANOVA, * *p* < 0.05; ** *p* < 0.01).

Under normal conditions, there was no difference in any physiological parameters between wild type (WT) and transgenic lines (Figure 11). Under drought and salt stress conditions, all physiological indexes showed a rising trend, but the trends differed. For example, after drought and salt stresses, the accumulation of MDA content in the leaves of transgenic *Arabidopsis* was significantly higher than that of wild type (WT), whereas the proline content, and the activities of POD, CAT, and SOD, were lower. These results further support that *TaERF-6-3A* is a negative regulator of drought and salt stress responses (Figure 11).

**Figure 12:**
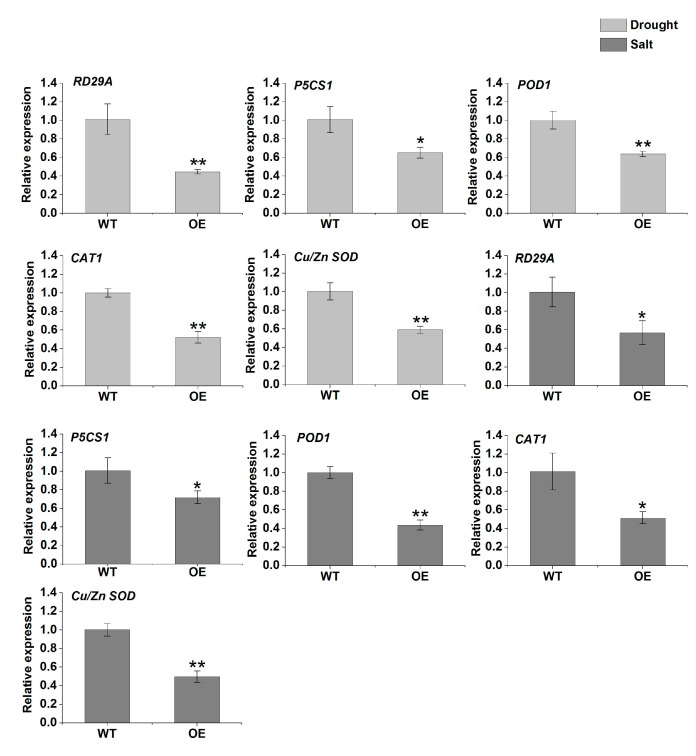
The expression profiles of stress-responsive genes (*RD29A*, and *P5CS1*) and three antioxidant-related genes (*POD1*, *Cu/Zn SOD* and *CAT1*) in *TaERF-6-3A* transgenic *Arabidopsis* lines under drought and salt stress. Standard deviations are indicated by error bars (mean ± SD and n = 3) and significant differences are indicated using asterisks (ANOVA, * *p* < 0.05; ** *p* < 0.01).

To elucidate the possible regulatory mechanism of *TaERF-6-3A* under abiotic stresses, we selected two known stress-related genes (*RD29A*, and *P5CS1*) and three antioxidant-related genes (*POD1*, *CAT1* and *Cu/Zn SOD*) in *Arabidopsis* for further investigation. Compared with the WT lines, the expression levels of the five genes were reduced in the overexpression lines under drought and salt stresses (Figure 12). The results indicate that the overexpression of *TaERF-6-3A* reduces drought and salt tolerance by directly or indirectly affecting the expression of abiotic stress- and antioxidant-related genes.

### 2.9. Transcription Activity and Yeast Two-Hybrid (Y2H) Analysis of TaERF-6-3A

**Figure 13:**
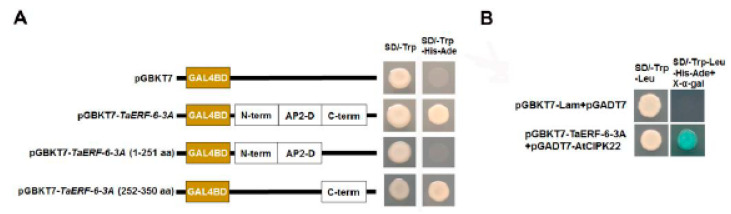
Transcriptional activity and yeast two-hybrid analysis of TaERF-6-3A. (**A**) Transcription activity analysis of TaERF-6-3A. The yeast cells were plated on SD/-Trp and SD/-Trp/-Ade/-His media. AP2-D, AP2/ERF domain; (**B**) Analysis of the interaction between TAERF-6-3A and AtCIPK22 in yeast.

To further investigate the potential molecular mechanism of *TaERF-6-3A*, we screened for proteins that might interact with TaERF-6-3A using the GeneMANIA database and uncovered a potential interacting protein named calcineurin B-like interacting protein kinase 22 (AtCIPK22) (Figure S4). First, we demonstrated that TaERF-6-3A had transcriptional activation activity and that the C-terminus region acted as a transcriptional domain (Figure 13A). We then performed yeast two-hybrid validation using the TaERF-6-3A as bait and AtCIPK22 (AT2G38490) as prey. The results show that TaERF-6-3A interacted with AtCIPK22 in the yeast two-hybrid system (Figure 13B).

## 3. Discussion

The AP2/ERF family is one of the largest families of plant transcription factors and plays an important role in the response to abiotic stress processes in *Arabidopsis* [20], rice [26], pepper [27], maize [23], and poplar [28]. Nevertheless, this is a detailed study concerning the structures, functions, and regulatory mechanisms of these genes in wheat. Our study using RNA-seq data before and after drought stress revealed that 20 *TaAP2/ERF* DEGs are present in wheat. The 20 TaAP2/ERF TFs were divided into four categories, AP2; ERF; DREB; and RAV. Unlike the AP2 family members, other subfamily members had almost no introns in terms of gene structure; the case is similar for the AP2/ERF family in sugarcane [29]. Previous study has shown that the number and distribution of introns is controlled by plant evolution [30], and some AP2/ERF families may lose introns in the course of plant evolution, suggesting that there may have been extensive differentiation and functional deviation among these subfamilies during the evolution of the wheat AP2/ERF family. In addition, identical and diverse motifs were found within and among TF groups, also supported the abovementioned suggestion. Motifs 1 and 2 are widespread among AP2/ERF family members and were identified in most of the AP2/ERF domains (Figure S3). Motif 1 was in the AP2/ERF domains of the majority of family members, even in WLG and RAYD. Motif 2 was retained in YRG (Figure S3). The amino acids in positions 14 and 19 of the AP2 domains of ERF (14A, 19D) and DREB (14V, 19E) are conserved and play an important role as target-binding DNA sequences [10]. Moreover, the changes in the two positions had different effects on DNA-binding ability. For example, when 14V in the AP2 domains of *DREB* genes was changed to 14A (A, alanine), the DNA-binding ability was drastically reduced, whereas the substitution of E with D (D, aspartic acid) at position 19 had little effect. Sakuma et al. found that the 14th amino acid of DREBs was always valine (V), but the 19th amino acid was not always glutamate (E), whereas the amino acids at positions 14 and 19 of ERFs were not always A and D, respectively [10]. In this study, the amino acids’ positions 14 and 19 of ERF TFs were not completely conserved, whereas DREB TFs showed complete conservation of the amino acid at position 14V and 19V (not always E) (Figure S3). The results of this study are consistent with those of previous studies [10] and suggest that ERF/DREB TFs in the same subfamily differ in their ability to bind downstream target genes.

Firstly, qRT-PCR was used to verify the expression of 20 genes under drought stress, and the results were generally consistent with the data of the transcriptome database. Then, we randomly selected five genes (*TaERF-3-3B; TaERF-6-3A; TaERF-7-5D; TaERF-9-2B;* and *TaERF-5-4B*) from the ERF family for further analysis. Subcellular localization and transcriptional activity analysis showed that all localized to the nucleus and had self-activation activity; both results are consistent with the characteristics of transcription factors.

We selected an *ERF* gene, *TaERF-6-3A*, which is induced by drought, salinity, and cold; constructed an overexpression vector, and transformed the vector into *Arabidopsis*. Interestingly, we found that overexpression of *TaERF-6-3A* decreased tolerance to drought and salt stresses. When plants are subjected to drought and salt stresses, some reactive oxygen species (ROS) are rapidly produced and accumulated in plant cells, resulting in severe oxidative damage [31,32]. MDA is an important physiological index used to measure the degree of plasma membrane oxidation [31]. Proline is a common osmolyte that accumulates under drought and salt stress and is involved in maintaining protein stability [33]. An antioxidative enzyme system (POD, CAT, and SOD) can remove ROS produced in plants under abiotic stress, enabling plants to survive extreme environments [34]. Therefore, the abovementioned physiological indices related to drought and salt stress can be used to evaluate plant resistance to abiotic stress quickly and accurately. In this study, the MDA content was higher in transgenic lines than in the wild type (WT) after drought and salt stresses, whereas the activities of POD, CAT, and SOD were lower in the overexpression (OE) lines than those in the WT (Figure 11). Meanwhile, the expression levels of antioxidant-related genes (*POD1*, *CAT1*, and *Cu/Zn SOD*) were significantly inhibited in transgenic lines after drought and salt stresses (Figure 12). These results suggest that *TaERFF-6-3A* repressed the antioxidant enzyme system, thereby aggravating oxidative damage and reducing salt and drought resistance.

In addition to the physiological indexes mentioned above, the tolerance of drought and salt stresses is also related to the induced expression of abiotic stress-related genes. Previous studies have shown that some ERF family TFs have a repressive effect on their target genes [35,36]. For example, overexpression of *TaERF4* in *Arabidopsis* increases the sensitivity to salinity stress, through the repression of *AtNHX* expression [37]. Moreover, *RD29A* is a well-studied marker gene that enacts protective functions to guard against stress damage [38]. *P5CS1* can be induced by various stresses and plays an active role in proline synthesis [39]. The expression levels of stress-related genes (*RD29A* and *P5CS1*) in *TaERF-6-3A* overexpression lines were obviously lower than in wild type (WT) lines (Figure 12). Correspondingly, the proline content of transgenic OE lines was significantly lower than that of the WT (Figure 11). These results suggest that *TaERF-6-3A* affects proline synthesis, represses the expression of stress-related genes, and seems to be involved in abiotic stress through the ABA signal pathway.

To further clarify the functions of *TaERF-6-3A*, a protein association network was generated using the GeneMANIA database. In this association network were found mainly calcineurin B-like (CBL) and CIPK family proteins, among which the AtCIPK22 interacts physically with TaERF-6-3A (Figure S4), and the relationship between the two proteins was confirmed in a yeast two-hybrid system (Figure 13B). In addition, AtCIPK22 and AtCIPK11 were co-expressed in the association network (Figure S4). The CBL family proteins are sensors of Ca^2+^ signals and interact with CIPK in response to a series of abiotic stress responses by regulating downstream target TFs [40]. For example, AtCIPK11 negatively mediates drought resistance through Di19-3 TFs [40]. We have demonstrated that TaERF-6-3A negatively regulates abiotic stress. Based on the above results, we speculate that TaERF-6-3A might be regulated by CIPK22 in wheat, which means that TaERF-6-3A might be the target TF of CIPK22. However, further experiments are needed to confirm this.

## 4. Materials and Methods

### 4.1. Identification of AP2/ERF Family Genes

Previously, we established a drought-stress transcriptome using the wheat variety JW1 (selected from spring wheat varieties “Field” and “NB1”-hybrid isolated populations). Two-week-old wheat leaves were collected before and after drought stress and sent to the Beijing Biomarker Biotech Company for RNA extraction and RNA-seq analysis [41]. Transcriptome sequencing was performed on six JW1 wheat samples after 0 or 6 h drought stress, which produced 70.36 Gb of clean data. The raw read sequences were uploaded and placed in NCBI Sequence Read Archive (SRA) under accession numbers SRR16077950; SRR16077951; SRR16077959; SRR16077956; SRR16077957; and SRR16077958. Qualified RNA was processed for library construction. In order to ensure the quality of the library, Qubit 2.0 and Agilent 2100 were used to examine the concentration of cDNA and insert size (a library with concentration larger than 2 nM is acceptable). Deep Sequencing was then performed using the Illumina platform. The reads containing poly-n-homopolymers, adapters, and other low-quality reads were removed from the raw data, clean reads were obtained for further analysis. The clean reads were aligned to the Chinese Spring wheat genome using HISAT2, and StringTie was applied to assemble the mapped reads. GATK3.2 and SnpEff3.6c were used for single-nucleotide polymorphism (SNP) analysis. Expression levels were calculated as fragments per kilobase of transcript per million fragments mapped (FPKM). Differentially expressed genes (DEGs) were assessed via the DEseq2 R package based on a negative binomial distribution model. The Benjamini–Hochberg method for controlling the FDR was used for adjustment of *P*-values. Unigenes with an adjusted *P*-value (set as false discovery rate (FDR) < 0.05 and Fold Change (FC) ≥ 2) were defined as differentially expressed genes DEGs. Volcano Plot was obtained using the Biomark Cloud Platform. Predicted protein sequences of DEGs from RNA-seq database were used to construct a local protein database. The HMM profile (ID: PF00847) of the AP2/ERF family was downloaded from PFAM database (http://pfam.xfam.org, accessed on 12 January 2022) and used for search and comparison operations in a local wheat database using HMMER3.0. Putative wheat AP2/ERF sequences containing at least one AP2/ERF domain were retrieved from the RNA-seq database of JW1 and used for the AP2/ERF family gene identification in this study. Thereafter, the candidate AP2/ERF proteins were confirmed by NCBI-CDD (https://www.ncbi.nlm.nih.gov/Structure/cdd/wrpsb.cgi, accessed on 17 March 2022). Finally, the molecular weight, number of amino acids, and theoretical isoelectric point of each TaAP2/ERF protein were predicted via ExPASy (https://www.expasy.org/, accessed on 12 January 2022). The subcellular locations of TaAP2/ERF proteins were predicted using CELLO (https://cello.life.nctu.edu.tw/, accessed on 12 January 2022).

### 4.2. Phylogenetic Analysis

All known AP2/ERF sequence data in *Arabidopsis* (TAIR10), and rice (IRGSP 1.0) were downloaded from Ensembl Plants (http://plants.ensembl.org/index.html, accessed on 12 January 2022). Multiple sequence alignment of the obtained AP2/ERF protein sequences was performed by ClustalX with default parameters. The neighbor-joining (NJ) algorithm was applied to construct phylogenetic trees using MEGA 7 software (https://www.megasoftware.net, accessed on 12 January 2022). The following parameters were applied: Bootstrap 1000 replicates, Poisson model, and pairwise deletion.

### 4.3. Chromosome Distribution, Gene Structure, and Conserved Motif Analyses

The chromosomal position data of the identified genes were acquired from the wheat genome annotation information (http://plants.ensembl.org/Triticum_aestivum/Info/Index, accessed on 12 January 2022). To visualize the homologous copies, lines among related genes were drawn using Circos software (http://circos.ca/software/, accessed on 12 January 2022). The gene structure file (the coding sequences (CDS) and genomic sequences) was retrieved from RNA-seq database, and the exon/intron structures were displayed using GSDS 2.0 (http://gsds.gao-lab.org/, accessed on 12 January 2022). Protein motifs were analyzed using MEME (https://meme-suite.org/meme/index.html, accessed on 12 January 2022).

### 4.4. Promoter Cis-Acting Elements Analysis

Genomic sequences corresponding to the regions approximately 2000 bp upstream of the *TaAP2/ERF* genes were uploaded to PlantCARE (http://bioinformatics.psb.ugent.be/webtools/plantcare/html/, accessed on 12 January 2022) to predict the *cis*-regulatory elements related to transcription, development, hormone, and abiotic stress.

### 4.5. Expression Profiling of TaAP2/ERF Genes by RNA-Seq Sequencing

The *TaAP2/ERF* gene fragments per kilobase million (FPKM) values of RNA-seq data were calculated and visualized by TBtools (https://github.com/CJ-Chen/TBtools/releases, accessed on 12 January 2022).

### 4.6. Plant Material and Stress Treatment

Wheat variety JW1 seeds were disinfected in 70% alcohol, then washed using sterilized water. The seeds were incubated in half-strength Hoagland solution in an artificial climate incubator (70% humidity, 18,000 Lux light intensity) under controlled conditions (20–25 °C, light/night, 16 h/8 h cycle). Two-week-old seedlings were subjected to drought (20% PEG6000), salt (200 mM NaCl), and cold (4 °C) stress treatments. Leaves were collected at 0, 1, 6, 12, and 24 h; the leaves treated at 0 h served as templates for gene cloning and as controls for the stress treatments. All samples were immediately stored at −80 °C for further mRNA extraction.

### 4.7. RNA Extraction and Gene Expression Analysis

Total RNA was extracted using the TRIzol reagent (TianGen, Beijing, China), and first-strand cDNA was synthesized with a reverse transcription kit (Transgen, Beijing, China), according to the manufacturer’s protocol. Quantitative real-time polymerase chain reaction (qRT-PCR) was performed on the Biosystems 7500 Fast Real-Time PCR System using the SYBR^®^ Green I reaction kit (Transgen, Beijing, China). Reactions of 20 μL contained 2 × TB Green SuperMix 10 μL, 50 × ROX Passive Reference Dye II 0.4 μL, 0.8 μL of each forward or reverse primer (10.0 μmol/L), 2 μL of cDNA (100 ng/μL), and RNase free water 6 μL. The qRT-PCR was conducted at 95 °C for 30 s, followed by 43 cycles of 95  °C for 5 s and 60 °C for 30 s. The fold change was calculated using the 2^−ΔΔCt^ method with wheat internal reference gene. Each qRT-PCR was conducted in three biological replicates. The details of the gene-specific primers that were used are shown in Table S2.

### 4.8. Gene Cloning TaAP2/ERFs in Wheat

The cDNA of 5 *TaERF* genes (*TaERF-3-3B; TaERF-6-3A; TaERF-7-5D; TaERF-9-2B;* and *TaERF-5-4B*) were amplified using specific primers (Table S2). Their PCR products were separately introduced into pEASY-T1 cloning vectors (TransGen, Beijing, China) and transformed into DH5α competent cells (Vazyme, Nanjing, China). The positive clones were then identified by PCR and sequencing.

### 4.9. Subcellular Localization Assay

The primers amplified the open reading frame (ORF) sequences of five TaERFs (*TaERF-3-3B*; *TaERF-6-3A*; *TaERF-7-5D*; *TaERF-9-2B;* and *TaERF-5-4B*) without a stop codon (Table S2); the PCR products were separately cloned into the *Bst*BI site of Pcambia1302-GFP vectors (CaMV 35S promoter). The fusion vectors were separately introduced into *Agrobacterium tumefaciens* strain GV3101, and the bacteria were injected into *Nicotiana benthamiana* leaves for transient expression. The results after 48 h of agro-infiltration were observed using a confocal laser scanning microscope (Olympus, Tokyo, Japan).

### 4.10. Transcriptional Activation Analysis in Yeast

The ORFs of five TaERFs (*TaERF-3-3B; TaERF-6-3A; TaERF-7-5D; TaERF-9-2B;* and *TaERF-5-4B*) were inserted into pGBKT7 vectors between *Eco*RI and *Sal*I restriction sites using the related primers, to construct recombinant vectors (Table S2), which were introduced into yeast for Y2H, to the test transcriptional activation of TaERF proteins. Cells with pGBKT7 were regard as the control group.

To research the transcriptional activation region of TaERF-6-3A, we truncated segments of TaERF-6-3A (1-251 aa and 252-350 aa), and they were fused to pGBKT7 to obtain different constructions, and then transformed into yeast Y2H (Table S2). We cultured on SD/-Trp and SD/-Trp/-Ade/-His deficient plates to determine whether there was self-activation.

### 4.11. Generation of TaERF-6-3A Transgenic Plants, Water Loss Rate, and Survival Rate Assay

The *TaERF-6-3A* coding sequence (CDS) sequence was cloned into a modified PBI121 expression vector between BamH I and Sac I restriction sites using primers (Table S2). The recombinant plasmid was transformed into wild type (WT) *Arabidopsis* Columbia-0 (Col 0) using the *Agrobacterium* (Strain GV3101) floral-dip method. The T_1_ transgenic *Arabidopsis* was screened on 1/2 MS plates (50mg/L kanamycin), transferred to soil, grown to maturity, and collecting T_2_ generation seeds. Individual T_2_ generation plants that segregated in 3:1 Mendelian ratio for kanamycin resistance were selected and homozygous T_3_ lines identified. Finally, twelve homozygous T_3_ transgenic lines were obtained.

To identify the resistance of salt and drought in *Arabidopsis*, three-week-old seedings of *TaERF-6-3A* transgenic *Arabidopsis* were given no water (12 days) and then re-watered (3 days) for drought survival rate analysis. Three-week-old seedlings were used for the water loss rate assay as well. *Arabidopsis* leaves similar in size and position were weighed for water loss and compared with the original fresh leaves. Three-week-old seedlings were supplied with 300 mM NaCl (15 days) for salt survival rate analysis.

### 4.12. Physiological and Biochemical Indexes and Stress- and Antioxidant-Related Gene Expression Analyses

The WT and OE *Arabidopsis* plants were collected for physical index measurements and stress-related gene expression abundance assessments after drought (10 days) and salt treatment (7 days). WT and transgenic lines were cultivated in the chamber at 25/19 °C (day/night), with 60% relative humidity, and 16 h light/8 h dark cycles. Malondialdehyde (MDA) content, proline content, and the activities of peroxidase (POD), superoxide dismutase (SOD) and catalase (CAT) antioxidant enzymes were measured as previously described [34,42]. Three biological replicates were carried out. The transcriptional levels of stress- and antioxidant-related genes were detected by qRT-PCR. The primers were listed in Table S2. Three biological replicates were conducted.

### 4.13. Prediction of a Candidate TaERF-6-3A-Interacting Protein

The GeneMANIA protein interaction database (http://genemania.org/, accessed on 12 January 2022) was used to predict the protein association network of TaERF-6-3A in *Arabidopsis*.

### 4.14. Point-to-Point Assay in a Yeast Two-Hybrid (Y2H) System

The details of the point-to-point experiment in yeast followed our previously research [43]. The recombinant plasmids containing pGBKT7-*TaERF-6-3A* and pGBKAD-*AtCIPK22* were transformed into Y2H and then cultured on SD/-Trp/-Leu and SD/-Trp/-Leu/-His/-Ade/+X-α-Gal solid media, respectively. The growth status and color response were observed after 2 to 3 days.

## 5. Conclusions

Our results clearly indicate that the stress-induced transcription factor TaERF-6-3A increases sensitivity to drought and salt stresses in transgenic *Arabidopsis*. This study also demonstrates the feasibility of searching for candidate genes involved in abiotic stress responses from transcriptome databases. These functions may be achieved by regulating the expression of relevant genes or their protein interactions. The next step should be to further elucidate the mechanisms involved in sensitivity tolerance in wheat, which will be the subject of future research.

## Data Availability

Not applicable.

## Supplementary Materials

The following supporting information can be downloaded at: https://www.mdpi.com/article/10.3390/ijms23063272/s1.
