# Supplementary material for: Transcriptomic Identification of Wheat AP2/ERF Transcription Factors and Functional Characterization of TaERF-6-3A in Response to Drought and Salinity Stresses
Source: Int J Mol Sci. 2022 Mar 18;23(6):3272. doi: 10.3390/ijms23063272 (PMC8950334; doi:10.3390/ijms23063272)
Supplement: Supplementary file 1 [file ijms-23-03272-s001.zip › ijms-1596388-supplementary/Supplemental Figures.pdf]

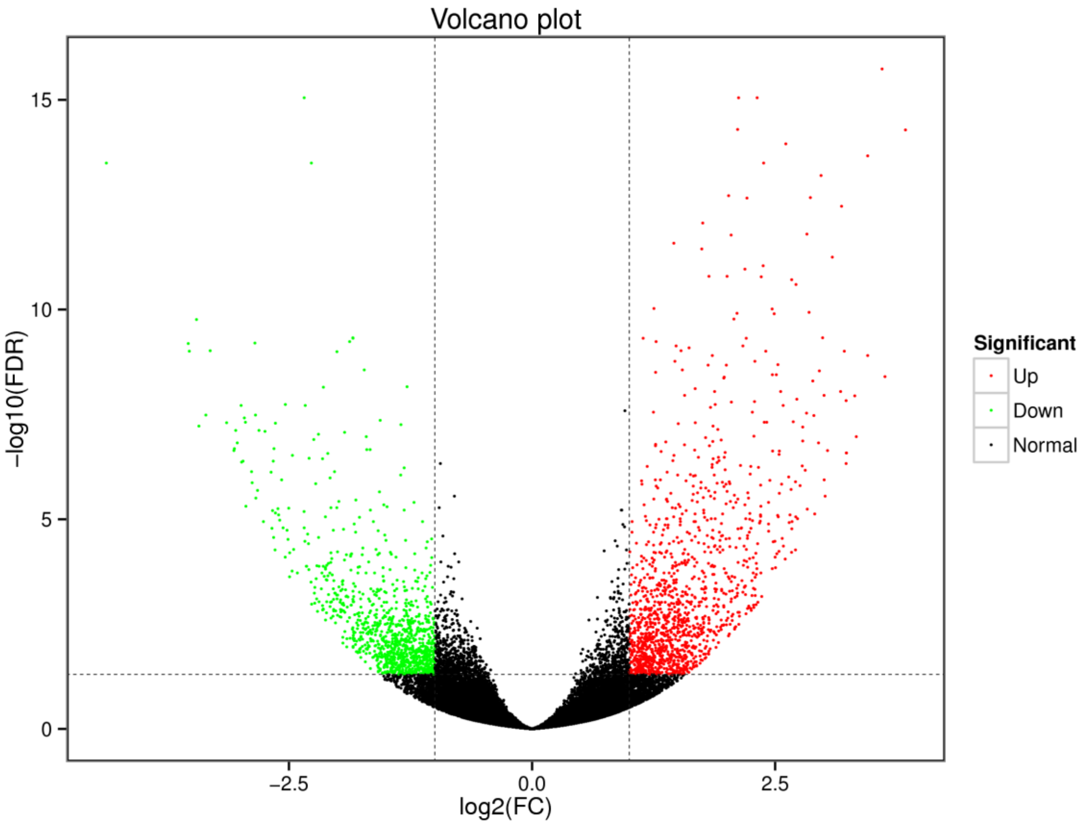

Supplemental Figure S1. Volcano plot of differentially expressed genes. Volcano plot represents the two vertical lines are the 2-fold change boundaries and the horizontal line is the statistical significance boundary ($p < 0.05$). Genes with fold change ≥ 2 and statistical significance are marked with green and red dots.

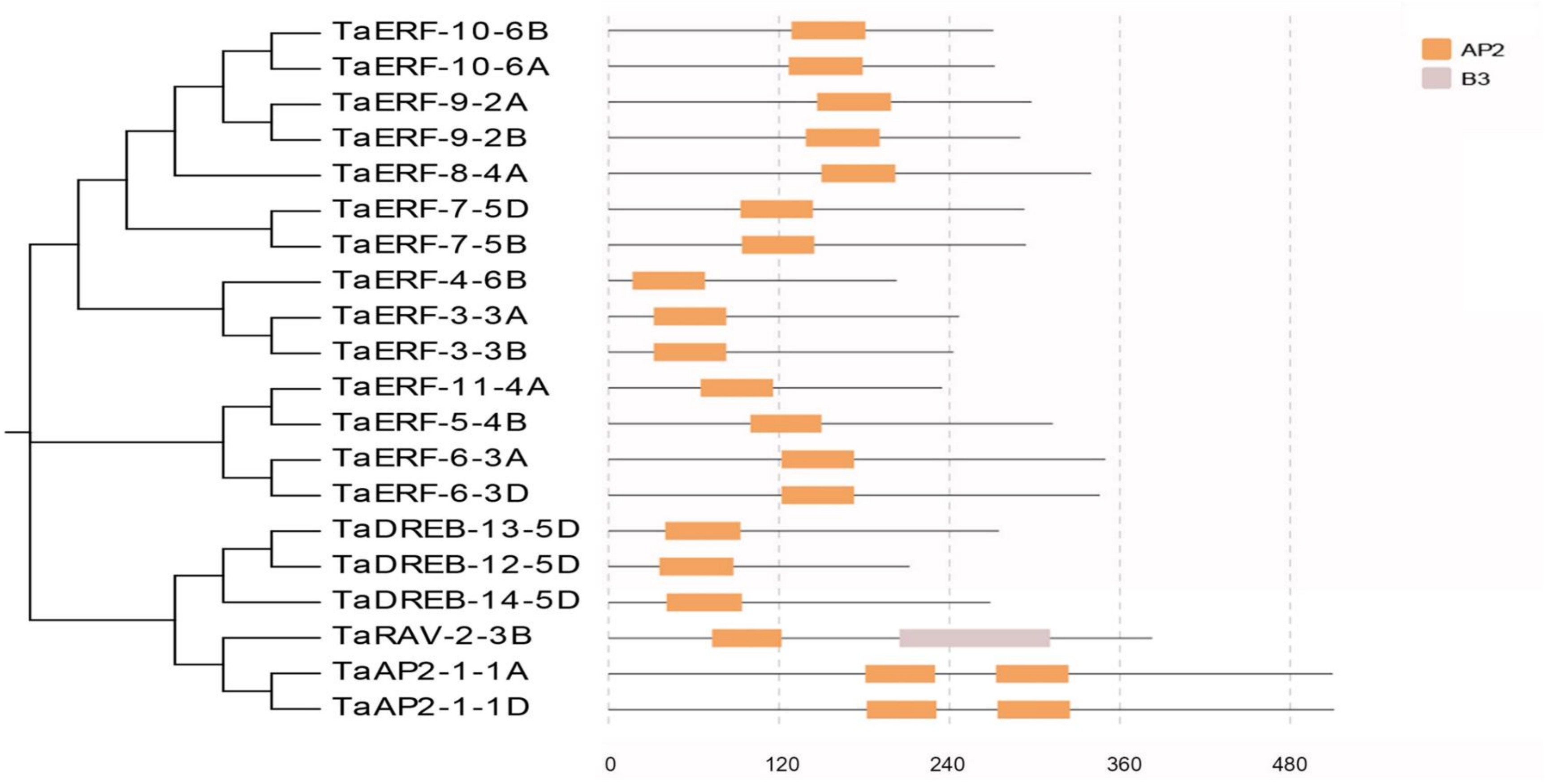

Supplemental Figure S2. The domain analysis of AP2/ERF gene. The different color boxes presented AP2/B3 domain.

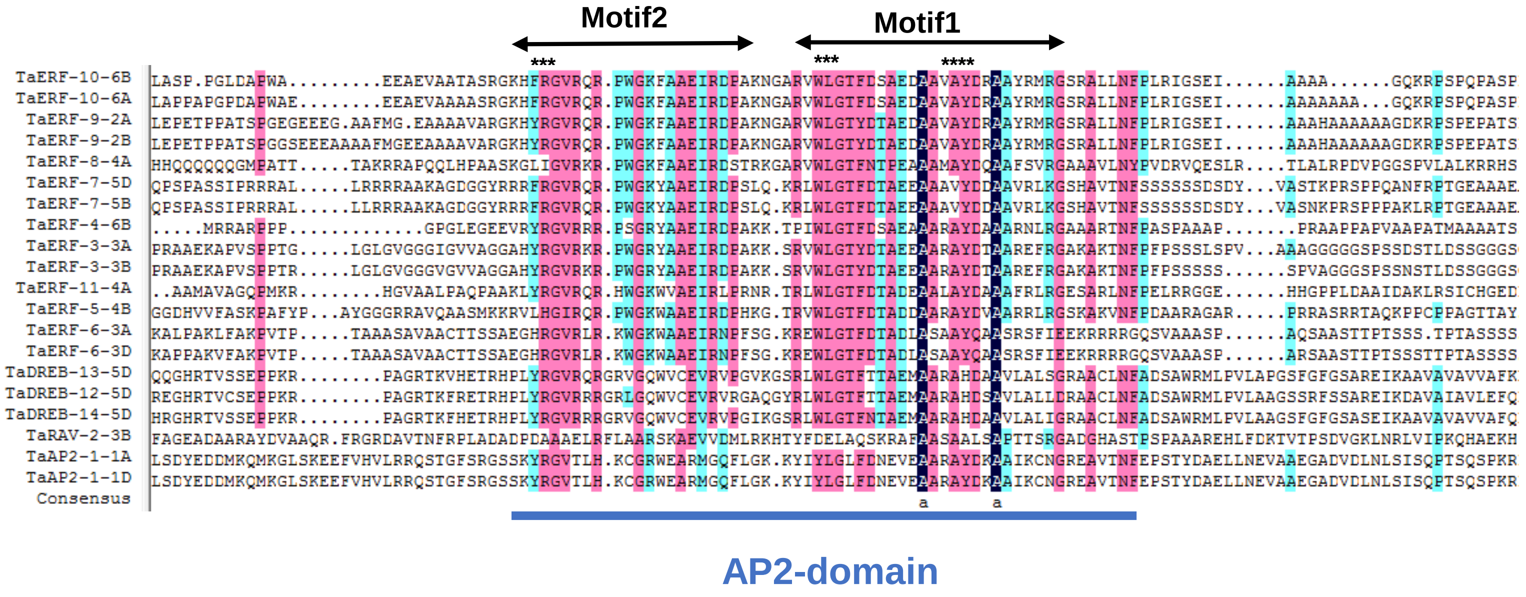

Supplemental Figure S3. Protein sequence alignments of the AP2/ERF domain. The blue line and green box represent the AP2 domain and EAR sequence.

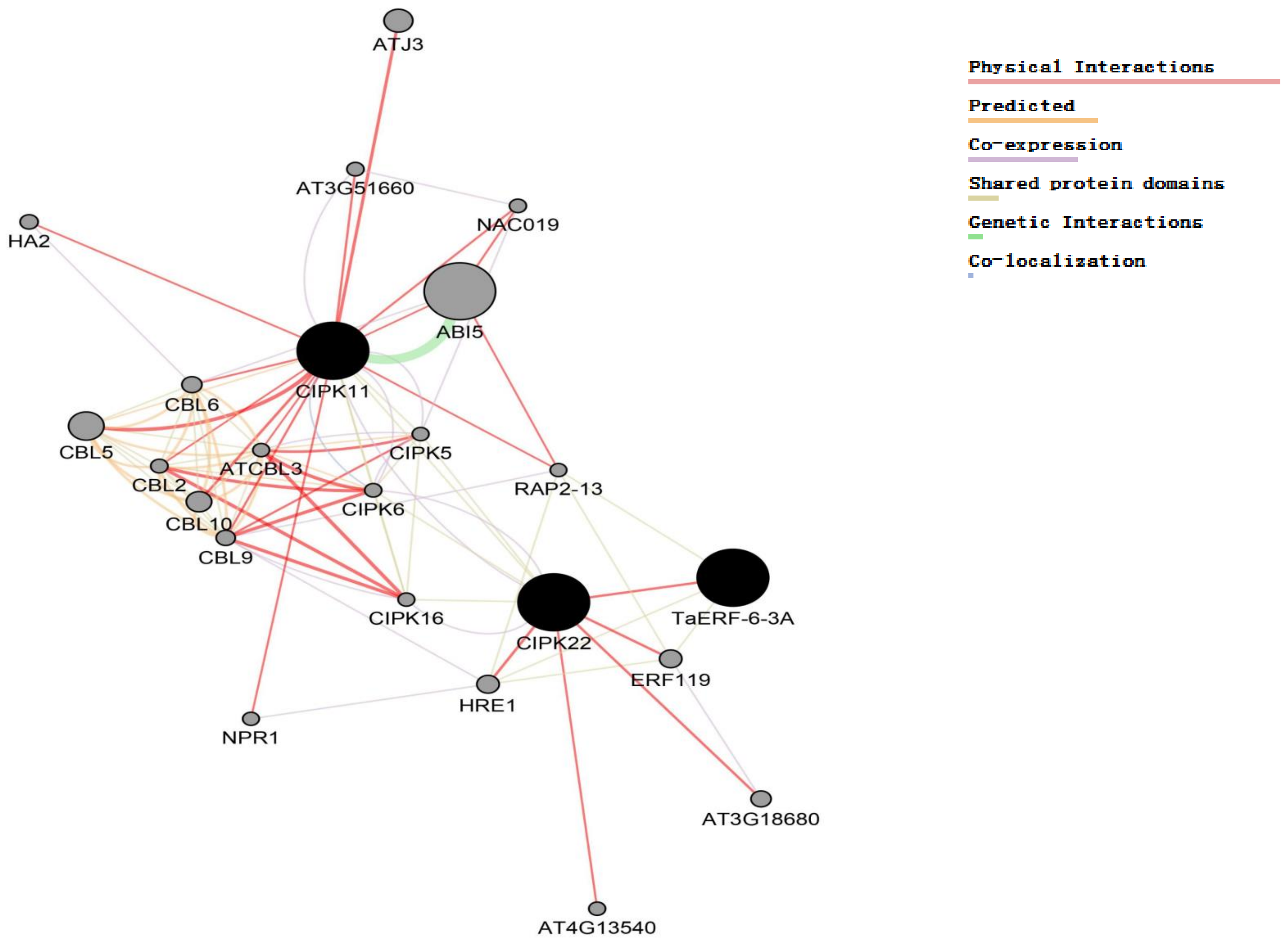

Supplemental Figure S4. Protein association network of TaERF-6-3A

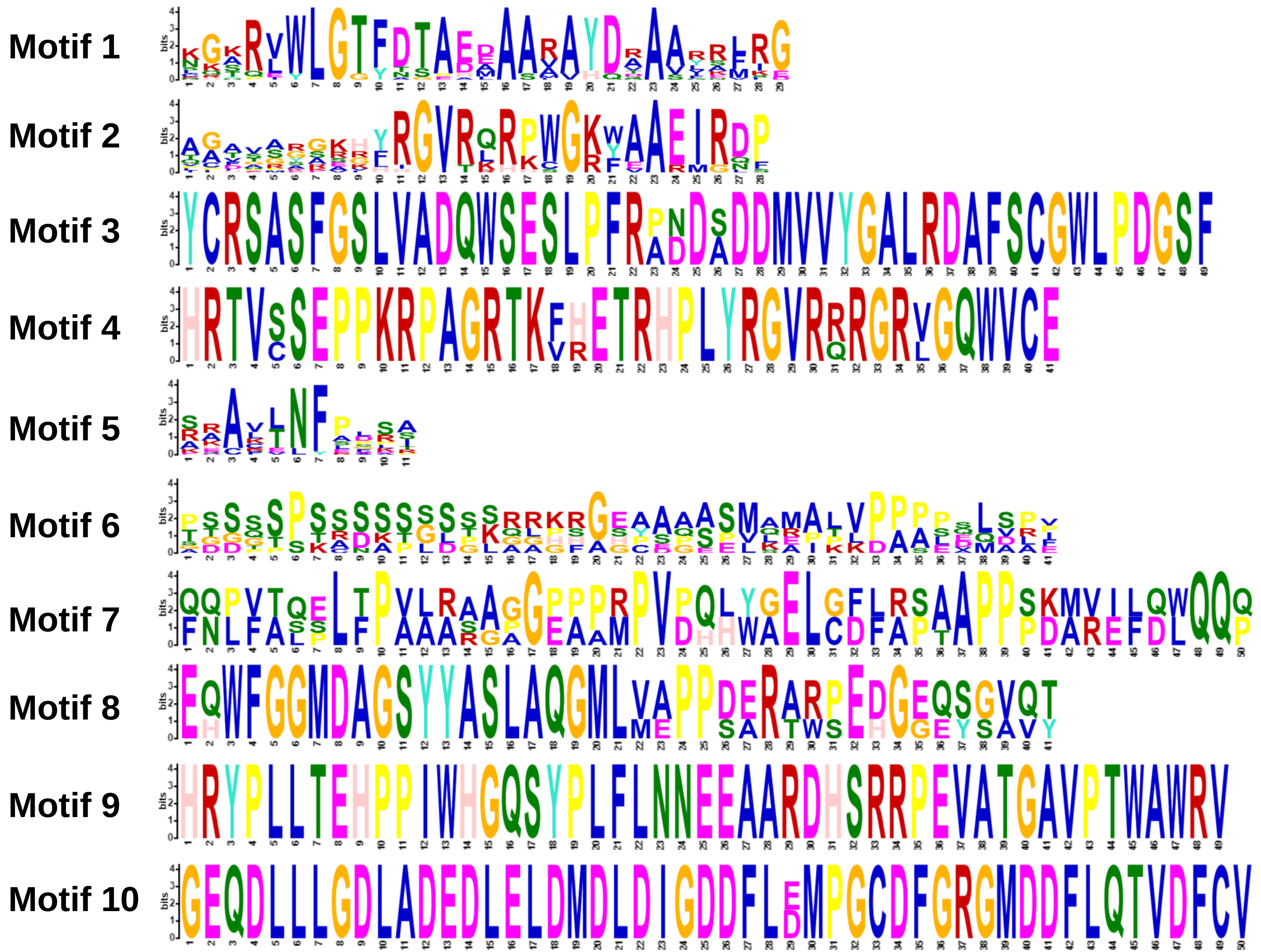

Supplemental Figure S5. Analysis and distribution of conserved motifs in wheat AP2/ERF proteins.
